# Aripiprazole-induced quasi-neuroleptic malignant syndrome: two case reports

**DOI:** 10.1186/s13256-024-04508-0

**Published:** 2024-04-18

**Authors:** Abdelgadir Hussein M. Osman, Joshua Wilkinson

**Affiliations:** 1 Faculty of Medicine, University of Khartoum, PO Box 201, Khartoum, Sudan; 2 Caludon Centre, Clifford Bridge Road, Coventry, CV2 2TE UK

**Keywords:** Creatine kinase, Neuroleptic malignant syndrome, Atypical antipsychotic

## Abstract

**Background:**

Significant elevation of creatine kinase levels (above three digits) and leucocytosis in the absence of muscle rigidity, tremors, or autonomic dysfunction can pose a real challenge in the context of antipsychotic treatment as an early herald of neuroleptic malignant syndrome.

**Case presentation:**

We present here two cases of adult male patients of Black British heritage, ages 51 years and 28 years, respectively. Both received a diagnosis of schizoaffective disorder and presented with massive increase of creatine kinase blood level after aripiprazole depot administration, one with pernicious increase associated with silent neuroleptic malignant syndrome, and the second with asymptomatic benign enzyme elevation.

**Conclusion:**

Though aripiprazole use is less likely to cause neuroleptic malignant syndrome, on rare occasions it can produce massive symptomatic or asymptomatic increase in serum creatine kinase enzyme levels, raising the need for close monitoring, especially at the initial doses of the drug.

## Background

Elevated creatine kinase (CK) activity can occur due to multiple reasons, including muscle injury, suspected rhabdomyolysis, intramuscular injections, heavy physical exercise, and cardiac and liver diseases. However, significant elevations in CK in patients treated with antipsychotics occur in neuroleptic malignant syndrome (NMS) [1, 2]. An increase in CK (over three digits) in the context of antipsychotic treatment can be considered a sign of NMS.

Atypical antipsychotics are known to be less incriminating in inducing NMS due to comparatively lower D2 receptor blocking potency. This is more so in the case of aripiprazole, as it is a partial D2 agonist, so does not have a total blocking effect [3]. Typically, NMS is known to present with fever, muscular rigidity, altered consciousness (confusion or rarely coma), disturbances in the autonomous nervous system (hypertension, tachycardia, tachypnoea, excessive sweating, and sometimes urinary incontinence) and deterioration in various blood values. This includes associated decreases in serum electrolytes, very high elevation in creatine phosphokinase (CPK), and leucocytosis. This acute clinical picture could suggest multiple potential infective and non-infective neurological differential diagnoses, however, the temporal relationship of the administration of the antipsychotic, combined with negative neuroimages and normal cerebrospinal fluid examination, is the usual pattern seen in NMS [4, 5].

The neurobiological mechanism underpinning the occurrence of NMS following the introduction of an antipsychotic is poorly understood, especially as to why individuals react in the way that leads to and generates the features of this syndrome. However, among the known risk factors of NMS, dopamine receptor blockade is found to be central to most theories of its pathogenesis [4–6]. For example, central dopamine receptor blockade in the hypothalamus may cause hyperthermia and other signs of dysautonomia. Another theory explaining the pathophysiology of NMS is an idiosyncratic reaction against certain antipsychotics, culminating in a cytotoxic effect that leads to the release of CK enzymes. This results in an immune reaction provoking leucocytosis, rigors, muscle contractions, and rigidity. Other components of NMS can be understood along the metabolic effects on the brain cells and neurons [4, 6, 7].

Researchers have examined a possible cytotoxicity and genotoxicity of aripiprazole in MKN45 and NIH3T3 cell in connection with cancer cells, which proved to have a beneficial effect. Therefore, it is no surprise that aripiprazole leads to huge release of CK enzymes due to cell destruction [7–9].

## Case report

### Case 1

Case 1 is a 51-year-old British male patient of Jamaican descent with an established diagnosis of schizoaffective disorder. He has had multiple psychiatric inpatient admissions and is also under the care of the community learning disability team but does not have a formal learning disability diagnosis. This current admission is his fifth inpatient admission over a 5-year period, in which he has previously been treated with both aripiprazole (oral tablets and depot) and quetiapine. His psychiatric medications on admission were sertraline 50 mg once daily, quetiapine modified release (MR) 400 mg every night and valproic acid 1 g twice daily.

An urgent outpatient appointment was arranged due to concerns from staff at his accommodation, who reported that he had been verbally aggressive, was shouting in the streets, partaking in a hunger strike, and threatening to kill staff members. His community mental health team had raised concerns that he was becoming more disruptive and was expressing delusional ideas, including a belief that he was being taken over by the devil.

A Mental Health Act Assessment was performed, and he was detained under Sect. 2 of the Mental Health Act. It was felt that he had relapsed in his mental health following noncompliance with his medications and illicit substance use.

Initially on the ward, he was verbally abusive, sexually inappropriate, and racist toward staff and other patients. He presented with pressured speech and grandiose and persecutory delusions, consistent with a manic episode, following a relapse of schizoaffective disorder. Quetiapine and sertraline were both discontinued and he was started on aripiprazole 10 mg twice daily, as he had previously responded well to aripiprazole during previous admissions. This was increased to 10 mg thrice daily after 3 days, before being switched to a monthly aripiprazole 400 mg depot. The first aripiprazole 400 mg depot was administered after 14 days treatment with oral aripiprazole.

During a clinical review 3 days after the aripiprazole 400 mg depot was given, he presented with new onset of delirium, so an urgent physical health assessment was performed including laboratory blood investigations. Physical examination and physical observations were unremarkable, but his creatine kinase was raised at 1311 U/L (reference range 46–171 U/L) and his inflammatory markers were elevated; white cell count 12.28 × 10^9^/L, neutrophils 9.76 × 10^9^/L, and C-reactive protein 32 mg/L. He had previously been treated with aripiprazole depot, but there were no previous creatine kinase levels to compare with. The main concern at this point was ruling out neuroleptic malignant syndrome. Following discussion with the medical team, he was transferred to the emergency department for further assessment but was discharged back to the psychiatric ward the same day.

Blood investigations were repeated the following day and his creatine kinase had now risen to 10,413 U/L, so he was subsequently transferred to emergency department again for further assessment. His blood tests were repeated in the emergency department, where his CK had increased further to 16,729 U/L. He was therefore admitted to a general medical ward for further assessment and management. He spent 4 days in an acute medical ward, where his creatine kinase levels improved, being 3652 U/L on the day of discharge (Table 1). No clear cause for the significantly raised CK levels was determined by the medical team, but it was felt that aripiprazole may be a contributing factor.

**Table 1:** Case 1’s creatine kinase level against time and changes in antipsychotic type

Time period	Creatine kinase level (U/L) (reference range 46–171 U/L)	Antipsychotic medication
Day 1 (day of admission)		
Day 2		Started on aripiprazole 10 mg twice daily
Day 5		Aripiprazole increased to 10 mg thrice daily
Day 16		First dose of aripiprazole 400 mg depot given
Day 19	1311	
Day 21	10,413	
Day 22	16,729	
Day 25	3652	
Day 30	2511	
Day 33	1101	
Day 44	283	
Day 47		Second dose of aripiprazole 400 mg depot given
Day 50	1288	
Day 51		Aripiprazole depot discontinued
Day 52	3870	
Day 62	213	
Day 68		Started on risperidone 2 mg every night
Day 71	134	risperidone increased to 4 mg every night
Day 86	210	
Day 96		Started on paliperidone depot
Day 112	124	

His mental state improved following the aripiprazole 400 mg depot, and he became more settled on the ward; 3 days prior to giving the second aripiprazole dose his creatine kinase levels were repeated and were 283 U/L, thus returning to normal levels. Therefore, he received his second dose of aripiprazole 400 mg depot 1 month (31 days) after the first dose and his creatine kinase levels were closely monitored; 3 days after receiving the second depot injection his creatine kinase had risen to 1288 U/L. He experienced no physical symptoms or any other adverse effects after his second depot injection apart from the rise in CK. CK levels continued to rise and were 3870 U/L 5 days after his second depot. Subsequently it was decided to stop aripiprazole as it was felt that the aripiprazole was causing the raise in CK levels. His antipsychotic medication was switched to risperidone and he was then started on paliperidone depot. His creatine kinase levels have remained in the normal range since.

### Case 2

Case 2 is a 28-year-old British Caribbean male with a diagnosis of autism spectrum disorder, with relapsing schizoaffective disorder. Often his relapses are triggered by acute stress. He has had multiple admissions to an inpatient setting, including three admissions in the past 12 months.

These admissions usually occur after a transient psychotic episode of affective components lasting for a few days, including paranoid ideations around friends and family, primarily triggered at times of distress. These episodes can lead to hospital admissions due to high levels of agitation and destruction of property, sometimes requiring involvement from the police.

During his most recent inpatient stay, his medications on admission were aripiprazole depot 400 mg monthly, depakote 500 mg every morning 1000 mg every night and diazepam 2 mg twice daily as needed. He was first started on aripiprazole 400 mg depot 5 years ago and previous notes indicate he has been maintained on aripiprazole since.

On routine blood investigations on a previous admission, his creatine kinase was raised at 1111 U/L and has stayed consistently raised since, as presented in Table 2. His only past level of creatine kinase was 4 years previously, which was also raised at that time, at 628 U/L (Table 2). He has remained asymptomatic with raised creatine kinase levels and has not complained of any physical symptoms while on aripiprazole. His physical observations, physical examination, and other blood investigation results have also been unremarkable. As he is asymptomatic, he remains on aripiprazole 400 mg depot monthly.

**Table 2:** Case 2’s CK level over time and with repeated administration of aripiprazole depot

Time period	Creatine kinase level (U/L)	Antipsychotic medication
Day 1 (day of previous admission)		
Day 4		Aripiprazole 400 mg depot given
Day 5	1111	
Day 11	1535	
Day 18	958	
4-month time frame; receiving aripiprazole 400 mg depot monthly		
Day		Aripiprazole 400 mg depot given in community
Day 14 (day of most recent admission)		
Day 15	1291	
Day 28		Aripiprazole 400 mg depot given
Day 32	1168	

## Discussions

Our two cases reported above exemplify an idiosyncratic reaction to aripiprazole with massive increase of CK levels. Notwithstanding a constant benign release of CK in the second case, this was not the case in the first case, where the massive increase to almost 30-fold was pernicious and associated with quasi-NMS. In comparison with what has mostly been reported elsewhere, elevation of CK is often benign and self-limiting following termination of the insulting agent.

We cited a few case reports of massive elevations in serum creatine kinase following the introduction of an atypical antipsychotic, such as olanzapine or aripiprazole. Such cases, constitute a real challenge due to the elevation of CK and possible leukocytosis in the absence of other features of NMS, such as muscle rigidity, tremors, fever, and autonomic irregularity [2–4]. However, other reports were thought to be due to rhabdomyolysis. It is believed that rhabdomyolysis can be part of NMS and that atypical antipsychotic drugs may cause it without other overt features of NMS. One hypothesis for the underlying pathophysiology is because of the antagonistic activity of aripiprazole at 5-HT2A receptors that exist in adult skeletal muscle. It is possible that the reduction in the density or blockade of 5-HT2A receptors would compromise the uptake of glucose and might lead to changes in sarcolemma that increase its permeability to CK [7–9].

Reports of excessive elevation of CK is not restricted to a specific atypical antipsychotic, as we can cite cases in connection with the use of risperidone, clozapine, olanzapine, ziprasidone, quetiapine, and aripiprazole. However, very few case reports cited an increase of CK above 25,000.

## Conclusion

Massive elevation of CK, over 25-fold, raises serious concerns of neuroleptic malignant syndrome, which is a life-threatening condition with potentially fatal consequences. Aripiprazole is an atypical antipsychotic that normally has safe administration and rarely leads to NMS with typical pernicious reaction, but on occasion aripiprazole can lead to a massive asymptomatic creatine kinase elevation (MACKE) [4]. Therefore, careful monitoring of all blood results is required for both typical and atypical antipsychotics.

## Data Availability

Data can be requested from corresponding author.
